# 3D-bioprinted GelMA/gelatin/amniotic membrane extract (AME) scaffold loaded with keratinocytes, fibroblasts, and endothelial cells for skin tissue engineering

**DOI:** 10.1038/s41598-024-62926-y

**Published:** 2024-06-03

**Authors:** Zahra Pazhouhnia, Alireza Noori, Ali Farzin, Keyvan Khoshmaram, Mahdieh Hoseinpour, Jafar Ai, Marzieh Ebrahimi, Nasrin Lotfibakhshaiesh

**Affiliations:** 1 Department of Tissue Engineering, School of Advanced Technologies in Medicine, Tehran University of Medical Sciences, Tehran, Iran; 2 AstraBionics Research Network (ARN), Universal Scientific Education and Research Network (USERN), Tehran, Iran; 3 Department of Tissue Engineering and Applied Cell Sciences, School of Medicine, Semnan University of Medical Sciences, Semnan, Iran; 4 Material Engineering Department, Faculty of Engineering, Tarbiat Modares University, Tehran, Iran; 5 Department of Life Science Engineering, Faculty of New Science and Technologies, University of Tehran, Tehran, 1417935840 Iran; 6 Department of Stem Cells and Developmental Biology, Cell Sciences Research Center, Royan Institute for Stem Cell Biology and Technology, ACECR, Tehran, Iran; 7 Regenerative Medicine Group (REMED), Universal Scientific Education and Research Network (USERN), Tehran, Iran

**Keywords:** GelMA, Gelatin, Amniotic membrane extract, Skin regeneration, Biomaterials, Regenerative medicine, Tissue engineering

## Abstract

Gelatin-methacryloyl (GelMA) is a highly adaptable biomaterial extensively utilized in skin regeneration applications. However, it is frequently imperative to enhance its physical and biological qualities by including supplementary substances in its composition. The purpose of this study was to fabricate and characterize a bi-layered GelMA-gelatin scaffold using 3D bioprinting. The upper section of the scaffold was encompassed with keratinocytes to simulate the epidermis, while the lower section included fibroblasts and HUVEC cells to mimic the dermis. A further step involved the addition of amniotic membrane extract (AME) to the scaffold in order to promote angiogenesis. The incorporation of gelatin into GelMA was found to enhance its stability and mechanical qualities. While the Alamar blue test demonstrated that a high concentration of GelMA (20%) resulted in a decrease in cell viability, the live/dead cell staining revealed that incorporation of AME increased the quantity of viable HUVECs. Further, gelatin upregulated the expression of KRT10 in keratinocytes and VIM in fibroblasts. Additionally, the histological staining results demonstrated the formation of well-defined skin layers and the creation of extracellular matrix (ECM) in GelMA/gelatin hydrogels during a 14-day culture period. Our study showed that a 3D-bioprinted composite scaffold comprising GelMA, gelatin, and AME can be used to regenerate skin tissues.

## Introduction

Skin serves as a crucial barrier, protecting the body from external influences. Nonetheless, it is susceptible to injuries and illnesses, contributing to the widespread problem of skin diseases^1^. Skin disorders rank as the fourth most prevalent cause of disability globally, excluding mortality^2^. Skin damage presents a substantial hurdle, impacting millions of individuals with severe burns and resulting in hundreds of thousands of deaths annually. While minor injuries can heal naturally, deeper lesions that pierce both the epidermis and dermis and necessitate medical intervention^3,4^.

Skin wounds are typically treated with autografts, allografts, or xenografts. Autografts are preferred due to their ability to promote wound healing, but they have limitations like donor site complications and limited availability^5^. Allografts eliminate the need for a graft from another bodily location, but they come with complications such as disease transmission, immune response, and insufficient viable cells^6,7^. Xenotransplantation, which involves using skin substitutes from animals such as pigs, is a low-cost therapeutic option, but a major concern with this approach is the rapid rejection of the graft^8^.

Tissue engineering has emerged as an alternative approach to the aforementioned procedures, utilizing living cells, biocompatible materials, and growth factors to construct skin-like structures^9^. The injured site receives these structures to enhance wound healing and tissue function. The design of the scaffolds used in tissue engineering is crucial for generating a suitable skin substitute^10,11^. A variety of common procedures, including solvent casting/particulate leaching^12^, gas foaming^13^, freeze drying^14^, and electrospinning^15^, have been implemented to produce scaffolds for skin tissue engineering. These processes are simple and economical, but they have a fundamental disadvantage in that they do not provide exact regulation over the microstructure of the produced scaffolds. Unlike the aforementioned traditional methods, rapid prototyping entails a collection of scaffolding techniques that use a computer program to build an object layer by layer. These methods can ascertain the internal and external structure of scaffolds, enhance the precision of processes, and automate manufacturing. The scaffolding design is based on virtual models or advanced medical imaging techniques such as CT scans to match the patient’s injured area. Extrusion printing is a type of printing technology that involves the use of a screw, piston, or air pressure to extrude ink from a nozzle. The fundamental advantage of this process is that it does not require laser light or heat, and it eliminates the need for harmful organic solvents. Nonetheless, it is crucial to optimize the viscosity of the ink for printing, and each printed layer must be capable of supporting its own weight.

Mimicking the layered structure of natural skin, researchers have developed multilayered scaffolds with keratinocytes in the upper layer to represent the epidermis, and fibroblasts in the lower layer mimicking the dermis^16^. Endothelial cells are occasionally positioned in the same region of the pseudodermis to enhance the formation of the vascular network^17^. 3D printing is a highly effective technique for creating such scaffolds. 3D bioprinting is popular for scaffold fabrication because it is customizable. In fact, 3D bioprinting can generates patient-specific scaffolds using imaging data from the injured area^18^. This technique employs rapid prototyping to layer-by-layer print cells, growth factors, and other biomaterials to create biomedical constructs that replicate the natural tissues^19,20^.

In addition to the scaffolding construction method, the composition and configuration of the scaffolds are also critical aspects. A diverse array of natural polymers, such as collagen, gelatin, and chitosan, along with synthetic polymers like polylactic acid, polyglycolic acid, and polycaprolactone, has been employed in fabricating skin tissue engineering scaffolds^11^. These polymers are employed in various formats, including mats, sponges, pre-made porous constructs, and hydrogels. Among the various types of scaffolds, hydrogel (water-swollen polymeric networks) has attracted significant interest due to its high water content, which mimics the original extracellular matrix (ECM) and can provide an optimal environment for cell survival. Additionally, hydrogels exhibit notable flexibility, biocompatibility, and wound healing capability^21^. Hydrogels can be prepared through physical or chemical cross-linking processes, which determine their characteristics and potential applications^22,23^. Gelatin and gelatin methacryloyl (GelMA) hydrogel are types of hydrogel that have demonstrated remarkable potential in tissue engineering and wound healing due to their ability to support cell activity and tissue development^24,25^. Although GelMA has advantageous features, using it in its pure form presents certain obstacles. GelMA has suboptimal printability, stability, and mechanical capabilities at low concentrations (< 10%, w/v)^16,26^. Conversely, GelMA in excess of 15% (w/v) concentrations substantially improves the compressive modulus, but cell adhesion and growth are negatively affected^26,27^. In an effort to enhance GelMA’s physical properties, it has been combined with other hydrogels such as silk fibroin^28^, cellulose^16^, and polyvinyl alcohol/sodium alginate^29^. Nevertheless, limited attention has been devoted to the development of a bi-layered scaffold for skin regeneration employing pure gelatin-based hydrogels. So, in the present study, we incorporate gelatin into GelMA to preserve or potentially improve its exceptional biological properties while also ensuring satisfactory physical and rheological characteristics at low concentrations.

Angiogenesis is another important factor in skin wound repair^30^. Various strategies have been explored to promote angiogenesis in wound healing, including the use of endothelial cells and angiogenic factors. In this context, the administration of amniotic membrane extract (AME), which contains angiogenesis-inducing components that accelerate the healing process, has gained attention. It is worth noting that the amniotic membrane (AM) is harvested from the deepest layer of the placenta and is commonly used in cosmetic surgery to treat skin injuries^31^. Subcutaneous injection of AME into the skin flap of rats has demonstrated an increase in angiogenic indicators, specifically new vessel counts and CD31 + levels, compared to controls^32^. Additionally, Kang et al. discovered that treating human fibroblasts with AME resulted in a 54.9% improvement in wound closure compared to the control group in a cell migration experiment. In a full-thickness skin wound model using rabbit ears, they also observed that wound sites treated with AME showed a well-formed epidermal basal cell layer 36 days after the injury. In contrast, wound sites that were not treated showed excessive cell growth, a damaged epidermis, and clumped collagen deposits in the newly formed skin^33^.

The aim of this study was to create a two-layer bioprinted scaffold using GelMA/gelatin/AME. The upper layer would consist of keratinocytes, while the lower layer would contain fibroblasts and HUVECs. The hypothesis was that adding gelatin to GelMA would improve its physical and biological properties, and the inclusion of AME would stimulate angiogenesis.

## Materials and methods

The present study is centered on the preparation and evaluation of 3D bioprinted gelatin/GelMA scaffolds for skin regeneration. It has been authorized by the ethical committee of Tehran University of Medical Sciences (IR.TUMS.VCR.REC.1398.972) and is in accordance with the editing and publication policies of the scientific reports journal. All procedures were carried out in compliance with the applicable rules and regulations.

### Synthesis and characterization of GelMA

In accordance with described protocol^28^, GelMA was synthesized. Briefly, gelatin (Type B, 225 bloom from bovine skin, Sigma-Aldrich, USA) was added to carbonate-bicarbonate buffer (50 ml, 0.25 M, pH = 9) at room temperature and agitated until completely dissolved. Then, 8 ml of methacrylate anhydride (MA) (purity: > 94%, CAS No. 760-93-0, Sigma-Aldrich, USA) was gradually added to the solution, and the reaction was allowed to proceed for 3 h at 55 °C. The reaction was then stopped by diluting it with 400 ml of Dulbecco’s phosphate-buffered saline (DPBS) (Gibco, Life Technologies, USA). Dialysis tubing cellulose membrane (12–14 kDa molecular weight cutoff, average flat width 43 mm, Sigma, USA) was used to remove salts and methacrylic acid from the solution for 7 days at 40 °C, with deionized water changed daily. The obtained GelMA was frozen at − 80 °C before being lyophilized.

1H NMR spectroscopy was used to confirm the methacrylation of gelatin. The 15 mg of gelatin or GelMA samples were completely dissolved in 1 ml of D_2_O water, supplemented with 0.05 wt% of 3-(trimethylsilyl)propionic-2,2,3,3-d4 acid sodium salt (Sigma-Aldrich, USA) as an internal standard. The spectroscopy was carried out at room temperature using a 400 MHz 1H NMR spectrometer (1H NMR, Bruker, Billerica, MA).

Further, to identify the functional groups present in the gelatin and GelMA, a Fourier-transform infrared (FTIR, Nexus 670, USA) analysis was conducted within the wavenumber range of 400–3600 cm^-1^, with a resolution of 1 cm^-1^.

### Preparation of the GelMA-based Inks

To make the prepolymer solution, lyophilized GelMA was dissolved in PBS at 80 °C in concentrations of 10%, 15%, or 20%w/v with mild agitation. Afterward, Irgacure 2959, Photoinitiator (2-Hydroxy-4′-(2-hydroxyethoxy)-2-methylpropiophenone, CAS. No. 106797-53-9, Sigma-Aldrich, USA), was added to the GelMA solution at a concentration of 0.25% w/v, and the mixture was agitated until the photoinitiator was completely dissolved.

To enhance the physical and biological properties of GelMA, different quantities of gelatin were integrated into the mixture. Based on preliminary testing, it was found that 15% w/v GelMA exhibited the best printability and biocompatibility. Consequently, this concentration was selected as the baseline for incorporating gelatin. Gelatin was dissolved in PBS and added to the GelMA/Irgacure solution at concentrations of 5%, 10%, and 15% w/v. As a result, subsequent investigations on the GelMA/gelatin composite encompassed three groups labeled as GelMA 15/gelatin 5, GelMA 15/gelatin 10, and GelMA 15/gelatin 15. The hydrogel polymerization occurred after 60 s of UV light irradiation (using EXFO Acticure 4000 with a wavelength of 365 nm).

### Characterization of GelMA/gelatin composites

#### Microstructure of the hydrogels

The morphological features of the produced hydrogels were analyzed using scanning electron microscopy (SEM; model: Gemini 300, ZEISS, Germany). Initially, the UV-crosslinked hydrogels were soaked in deionized water overnight at 37 °C. Subsequently, the hydrogels were frozen at − 80 °C and lyophilized for two days. A thin layer of gold was deposited onto the hydrogels, and the cross-sectional views of the samples were examined using the SEM. The pore size distribution of the samples was measured using ImageJ software (version 1.51J8; https://imagej.nih.gov/ij/).

#### Rheological Behavior

The rheological characteristics of the hydrogels were determined at 25 °C using a parallel plate model of a TA DHR-2 rheometer (TA Instruments, USA). Briefly, the sample was loaded onto the rheometer stage, and the steel plate was lowered until it made contact with the sample’s surface. Subsequently, plate was further lowered until the instrument’s axial force reached 0.02 N. The storage modulus (Gʹ) and loss modulus (Gʹʹ) were measured at a strain of 1% and an oscillation frequency of 1 Hz while the temperature was gradually reduced from 40 to 10 °C at a rate of 1 °C/min. To ascertain the correlation between the composite viscosity and shear rate of each group, the bioinks were scanned for 120 s at 15 and 25 °C, and at shear rates ranging from 0.001 to 100 s^-1^.

### Printing the scaffolds

After preparing the inks, an extrusion-based 3D bioprinter (3DBIO, Iran) was employed to fabricate the scaffolds. The printer, like other printing devices, was operated by a computer program. Computer-aided design software (AutoCad, version 2019; https://www.autodesk.com/products/autocad) was used to design a rectangular cube model (10 × 10 × 4.8 mm) for the scaffolding, which was then printed using Repetier-Host version 2.1.6 software (https://download3.repetier.com/files/host/win/setupRepetierHost_2_1_6.exe). In this model of device, the metal plate on which the printing takes place moves in the x and y directions, while the syringe holder, after printing an x–y plate, is elevated to the desired size by the controller in the z direction. The ink was administered using the 22G nozzle, which has an inner diameter of 0.5 mm, at a printing velocity of 20 mm/s. The thickness of each layer and the spacing between two strands were adjusted to 0.6 mm and 0.4 mm, respectively, while the substrate temperature was maintained at 4 °C. The printed structures were then exposed to ultraviolet light in a UV chamber for crosslinking.

#### Tensile test

The EZ test machine (Shimadzu, Kyoto, Japan) with a 200 kN load cell was used to determine the tensile strength of the specimens. The scaffold specimens were shaped like a dumbbell with a thickness of 2 mm. During the test, the scaffold was pulled at a constant rate of 1 mm/min until it torn completely, and the corresponding stress and strain were recorded. The Young’s modulus of specimens was estimated as the gradient of the stress–strain curve’s initial linear stage in the 0–10% strain range.

#### Swelling, and degradability of the hydrogels

To determine the equilibrium swelling ratio, the scaffolds, measuring 8 mm in diameter and 400 μm in thickness, were initially freeze-dried and weighed to measure their dry weight (W_d_). Afterward, these samples were immersed in 1.5 ml of PBS at 37 °C until they reached a state of balanced swelling. Following the careful removal of any surplus PBS from the sample using filter paper, the increased weight of each sample was meticulously recorded as W_s_. The degree of swelling (DS) ratio of the scaffolds was determined as follows:

$$DS\left( \% \right) = \frac{{W_{s} - W_{d} }}{{W_{s} }} \times 100$$

The in vitro biodegradability of the scaffolds was analyzed by soaking them in PBS containing 1.25 U/mL type II collagenase (C6885-500 MG, Sigma-Aldrich, USA) for up to 14 days at 37 °C. Prior to submersion in 1.5 mL of PBS, the initial weight of the scaffolds was recorded as W_0_. The submerging medium was refreshed every 3 days to maintain enzyme activity. At predetermined time points, the printed scaffolds were taken from PBS and freeze-dried after being washed with deionized water. Once the samples had dried, their weights were measured as W_t_. The degradation ratio of the 3D-printed scaffolds was calculated using the following formula:

$$Degradation\;Ratio \left( \% \right) = \frac{{\left( {W_{t} - W_{0} } \right)}}{{W_{0} }} \times 100$$

### Biological assessment

#### Preparation of amniotic membrane extract (AME)

The AME was prepared in accordance with the previously outlined procedure^34^. Human Amniotic Membranes (HAMs) were collected from the Amniotic Membrane Bank at Royan Institute in Tehran, Iran, which has ethical authority to bank these HAMs (EC/92/10/72). After being treated with antibiotics and a PBS solution, the HAMs were frozen in liquid nitrogen and crushed into a powder. To get rid of the debris, the powder was combined with water, homogenized, and centrifuged. The powders were then poured into PBS at a concentration of 0.1 mg/mL and dissolved after 4 h of agitation on the stirrer. After passing through a 0.2-µm filter, the product was kept at − 70 °C.

#### Cells behavior in the hydrogels

Human dermal fibroblasts (HDF, NCBI Code C645) and human umbilical vein endothelial cells (HUVEC, NCBI Code C554) were purchased from the Pasteur Institute of Iran, while the human-derived keratinocyte HaCaT cell line was kindly gifted by the Viracell Company in Iran. The obtained cells were cultured in a 6 cm tissue culture dish containing 8 mL of Dulbecco’s modified Eagle medium (DMEM; Gibco, USA) with 10% fetal bovine serum (Gibco, USA) and 1% (v/v) penicillin and streptomycin (Sigma, USA). The cells were maintained in a 5% CO_2_ incubator at 37 °C.

To culture the cells within hydrogels, keratinocytes, fibroblasts, and HUVECs were suspended individually in cell culture medium and mixed with 300 μl of GelMA pre-polymer solution. The ultimate cell concentration in the GelMA or GelMA-gelatin hydrogels was fixed at 0.5 × 10^5^ cells/ml. After pipetting the pre-polymer solution containing the cells, 100 μl of the hydrogel-cell suspension was placed into triplicate microwells in a 96-well plate and then subjected to UV crosslinking to generate a cell- encapsulated hydrogel. The metabolic activity of the cells enclosed within GelMA or GelMA-gelatin samples was assessed on days 1, 7, and 14 using the Alamar blue reagent (Invitrogen, Carlsbad, CA, USA) assay, following the directions provided by the manufacturer. At specified intervals, 10% v/v Alamar blue solution was added to the wells containing the hydrogels. The plate was then incubated at 37 °C for an additional 3 h. Afterward, 100 μL of the media was extracted and transferred to a new 96-well plate for analysis at 560/590 nm (Ex/Em) using a Spectramax plate reader (San Jose, USA).

Based on the results obtained from the Alamar blue and physical tests, the G15/g10 sample was selected for subsequent evaluations. The morphological features of proliferating HDF fibroblasts and HaCaT keratinocytes seeded on the hydrogels were assessed by means of SEM. After 4 days of culture, cells were fixed in 2.5% glutaraldehyde for 45 min at 4 °C. They were then rinsed and dehydrated using a succession of aqueous ethanol solutions ranging from 50 to 100% concentration. After additional vacuum drying, the samples were examined SEM in accordance with the procedures described in the Scanning electron microscopy section.

A tube formation test was carried out to investigate the angiogenic potential of hydrogels as well as the effect of AME on this capacity. The GelMA15/g10 hydrogel group was supplemented with AME at a concentration of 0.1 mg/ml. In brief, 300 µL of Matrigel or GelMA15/g10 was applied to each well of 24-well cell culture plates. Polymerization occurred within 30 min at 37 °C. Polymerization o the hydrogels occurred within 30 min at 37 °C. Afterward, 5 × 10^4^ of HUVECs were seeded in each wells. The capillary tube branch points produced by HUVECs were captured in five random microscopic fields per well using an inverted light microscope (Olympus, Japan) after 12 and 24 h of incubation.

The expression of the keratinocyte keratin 10 (KRT10), fibroblast vimentin (VIM), and HUVEC vascular endothelial growth factor (VEGF) genes was measured using real-time polymerase chain reaction (RT-qPCR), compared to the housekeeping gene glyceraldehyde-3-phosphate dehydrogenase (GAPDH). Following 14 days of incubation, cell-laden hydrogel samples were rinsed with PBS, shock-frozen in liquid nitrogen, and then crushed in microcentrifuge tubes. The cells’ total RNA was extracted using the GeneAll RiboEx RNA extraction kit (GeneAll Biotechnology, Korea) according to the manufacturers’ instructions. Subsequently, the concentration and purity of the RNA were assessed using a Thermo Scientific NanoDrop 1000 reader. Primers were designed with the Primer Express software (version 3.0.1; https://www.thermofisher.com/order/catalog/product/4363993#/4363993) (Table 1). Complementary DNA (cDNA) was synthesized from 1 μg of total RNA using a cDNA Synthesis Kit (Yekta Tajhiz, Iran) as per the manufacturer’s guidelines. Next, a 96-well PCR plate (Nest Biotechnology, Wuxi, China) was filled with 10 μL of each sample. The qRT-PCR analysis was performed utilising the SYBR Green Master Mix kit (Vazyme, China) and the 7900 real-time PCR system (Applied Biosystems, Foster City, CA, USA). The Ct values were obtained from three samples in each time point and in each group to determine the mean and standard deviation. The relative gene expression, in comparison to the reference gene GAPDH, was calculated.

**Table 1:** Primers sequences and PCR product size of *KRT10* and *VIM* genes.

Gene	Primer sequences (5^’^ > 3^’^)	Tm	PCR product size
*KRT10*	F:TCTAGCAGCAAAGGCTCCCT R:CCATAGCCACCTGATGAGCC	60^0C^	116 bp
*VIM*	F:GGACCAGCTAACCAACGACA R:AAGGTCAAGACGTGCCAGAG	60^0C^	178 bp
VEGF	F:TGCAGATTATGCGGATCAAACC R:TGCATTCACATTTGTTGTGCTGTAG	60^0C^	81 bp

#### Cells behavior in 3D-Printed GelMA

The bioprinting procedure aimed to precisely replicate human skin composition. Cells were encapsulated in GelMA/gelatin hydrogel, as detailed in the Alamar blue assay section, and then printed following the procedure outlined in “Printing the scaffolds” section. To mimic the epidermis, two layers of GelMA/gelatin containing keratinocytes were printed, each 0.6 mm thick. For the dermis simulation, six layers of GelMA/gelatin containing fibroblasts and HUVECs were printed, resulting in a total thickness of 3.6 mm. Further, the dermis region was administered 50 μL of PBS-dissolved AME. Finally, the separately printed epidermis and dermis sections were seamlessly joined together, facilitated by the strong adhesive characteristics of GelMA.

Based on the Alamar blue test outcomes, the GelMA15/g10 group was selected for further investigations. Cell viability in these hydrogels was evaluated using the LIVE/DEAD® Viability/Cytotoxicity kit (Sigma-Aldrich, USA), both with and without AME. HUVEC cells were enclosed within the hydrogels following the procedure outlined in the Alamar blue test section and then placed in a CO_2_ incubator at 37 °C. Simultaneously, the bioinks containing cells were printed as previously described, and after UV crosslinking, the scaffolds were placed at 37 °C in a CO_2_ incubator. On days 7 and 14, the hydrogels and scaffolds were retrieved from the culture, followed by a 20-min double-staining procedure utilising Calcein-AM (2 μM in PBS) and Propidium Iodide (4 μM) at ambient temperature (RT). After washing with PBS, the samples were examined using a Cytation 5 Imaging Reader (BioTek, USA). Image analysis was performed using ImageJ software to quantify the number of viable HUVECs.

Following both a 7- and 14-day culture period, the bioprinted scaffolds underwent rinsing with PBS and subsequent fixation at room temperature for 48 h using 10% neutral buffered formalin. Next, the samples underwent dehydration using a gradient of ethanol solutions ranging from 70 to 99.5%. Afterwards, the specimens were paraffin-embedded and sliced to produce 5 µm-thick cross-sections. Following this, the cross-sections underwent deparaffinization and were subjected to staining with hematoxylin and eosin dyes (H&E staining) for assessing cell morphology, as well as Masson’s trichrome (MT) staining for detecting collagen fibers. Optical microscopy was employed to examine the stained samples.

### Statistical analysis

In this study, the quantitative data were reported as means ± standard deviations (SD). Statistical analysis of multiple comparisons were conducted using Tukey’s post hoc test after performing a one-way ANOVA in GraphPad Prism version 7.0 (https://graphpad-prism.software.informer.com/7.0/). A *p* value less than 0.05 was denoted by a single asterisk (*), *p* < 0.01 by two asterisks (**), *p* < 0.001 by three asterisks (***) and *p* < 0.0001 by four asterisks (****).

## Results and discussion

The primary motivation in the field of skin tissue engineering is to achieve scar-free wound healing. In this study, 3D printing technology was employed to create a scaffold containing keratinocytes in its upper portion to simulate the epidermis, as well as fibroblasts and endothelial cells in its lower part to model the dermis.

### Synthesize and characterization of GelMA

**Figure 1:**
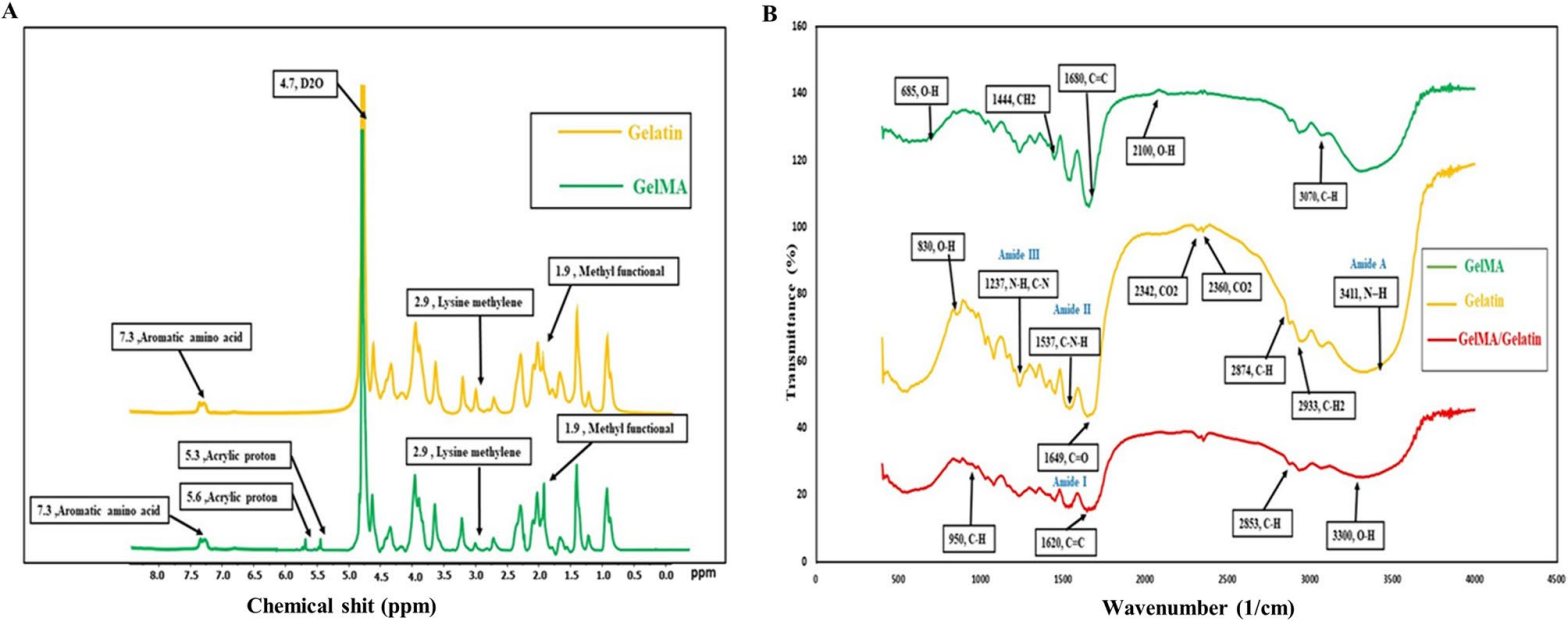
H-NMR spectrum of gelatin and GelMA **(A).** FTIR spectrum of gelatin, GelMA, and GelMA/gelatin **(B)**.

The addition of methacryloyl groups to gelatin was confirmed using 1H NMR analysis (Fig. 1A). In the GelMA spectra, new signals appeared compared to unmodified gelatin, indicating the presence of methacryloyl groups. Specifically, signal at 1.8 ppm was attributed to methyl function, while peaks at 5.3 ppm and 5.6 ppm were assigned to the acrylic protons of methacryloyl groups^26,35^. Further, the signal from the lysine’s methylene group at 2.9 ppm decreased in GelMA, suggesting the conversion of free amino groups of gelatin^36^. The results indicate the successful molecular-level alteration of gelatin through the exchange of functional groups such as amino and hydroxyl.

Figure 1B illustrates the FTIR spectra of gelatin, GelMA, and the gelatin/GelMA composite. The gelatin spectra display peaks at 1650, 1537, and 1237 cm^−1^, corresponding to the stretching of the C=O and C–N bonds (amide I), the bending of the N–H bond (amide II), and the stretching of the C-N and bending of the N–H bond (amide III), respectively. Additionally, the peaks at 2874 cm^−1^ and 2933 cm^−1^ are associated with CH and CH_2_ stretching vibrations, respectively, while the peak at 3411 cm^−1^ represents N–H stretching (amide A). Comparing GelMA and the GelMA/gelatin composite to gelatin, noticeable shifts and alterations are observed in their FTIR spectra, which can be attributed to the chemical processing involved in creating GelMA^35,37,38^.

### Physicochemical and biological characterization of GelMA\gelatin hydrogel

**Figure 2:**
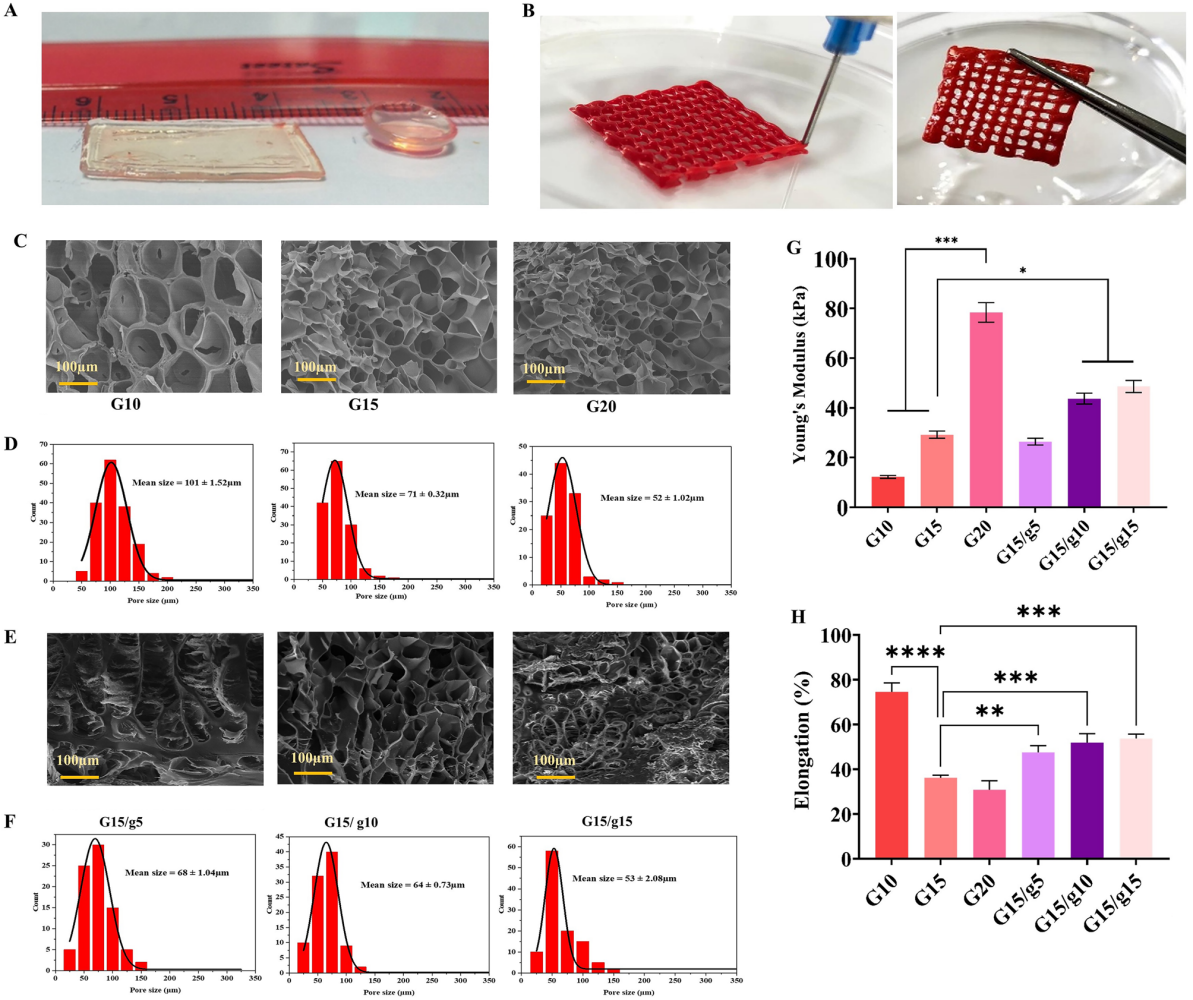
Macroscopic photographs of GelMA/gelatin hydrogel after UV crosslinking **(A)**. Macroscopic images of the scaffolds during and after the printing **(B)**. SEM micrographs of GelMA hydrogels at three concentrations **(C)** and GelMA/gelatin hydrogels **(E)** (scale bar = 100 μm). The pore size distribution graphs for the hydrogels were generated using ImageJ software (**D** and **F**). The mechanical properties of the GelMA and GelMA/gelatin hydrogels were assessed by measuring the Young’s modulus **(G)** and elongation at break **(H)**.

Figure 2A illustrates macroscopic photographs of GelMA/gelatin hydrogel after UV crosslinking, while the macroscopic images of the scaffolds taken during and after the printing process are displayed in Fig. 2B.

Hydrogel porosity, pore size distribution, and interpore connectivity impact cell behavior. The optimal pore size for cell penetration varies by cell type; however, a range of 20–125 µm is recommended for skin regeneration^39^. The SEM micrographs of the various groups of GelMA and GelMA/gelatin hydrogels are displayed in Fig. 2C,E. The size distribution of the scaffold pores, measured by the ImageJ software, is shown below each scaffold image (Fig. 2D,F). All three concentrations of GelMA hydrogels have very porous structures with varied pore shapes and sizes. Consistent with prior research, increasing GelMA solution concentration decreased hydrogel average pore size^26–28^. These macropores are separated by smooth, thin walls. According to reports, blood vessel formation, nourishment, and the transportation of metabolic waste are all hindered by tiny pore sizes, but surface area and cell attachment are reduced by large pore diameters^40,41^. As a result, the medium concentration of GelMA, 15%, was used to create the gelatin and GelMA composite.

Figure 2G illustrates that higher GelMA concentrations increase hydrogel Young’s modulus. The moduli range from 14.1 kPa for G10 to 78.4 kPa for G20, which can be ascribed to a higher crosslinking density at higher GelMA concentrations^42^. Moreover, adding 5% or 10% gelatin to 15% GelMA hydrogel increases its Young’s modulus significantly (*p* < 0.05), likely due to the formation of denser polymer networks in the GelMA structure ^16^. Materials with a firmer matrix may enhance keratinocyte growth. Further, hydrogels with greater elastic moduli may benefit implantations and surgical operations ^26^. Additionally, a higher GelMA concentration reduces elongation at break points from 74% (10% GelMA) to 30% (20% GelMA), perhaps due to greater crosslinking density restricting hydrogel deformation (Fig. 2H). Furthermore, adding gelatin to GelMA significantly increases its elongation at break. The GelMA15/g10 sample’s 56% extensibility is considered acceptable for skin substitutes, because it enables the material to withstand sudden stretching or bending, preventing premature failures after implantation^43^. Overall, the results showed that by modifying the polymer concentrations, it is possible to create gelatin/GelMA hydrogels with diverse mechanical characteristics that are appropriate for application in skin tissue engineering.

**Figure 3:**
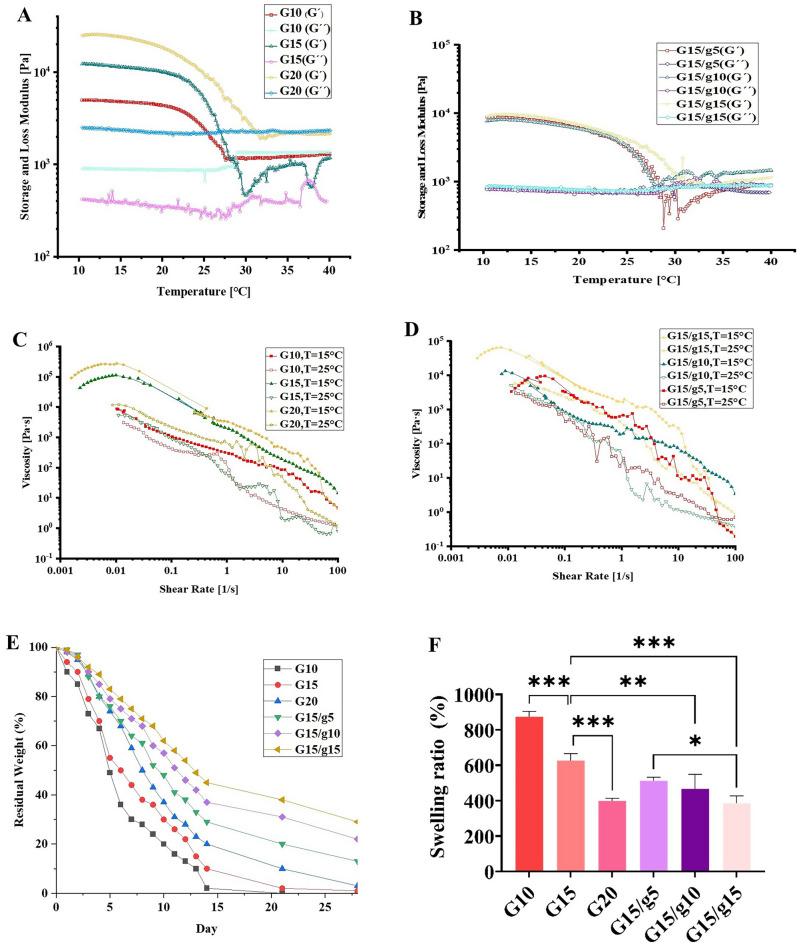
The Storage and Loss modulus of GelMA **(A)** and GelMA/gelatin hydrogels **(B)** as a function of temperature. Viscosity as a function of shear rate at 15 and 25 ^◦^C for GelMA **(C)** and GelMA/gelatin hydrogels **(D)**. Enzymatic degradation of the GelMA hydrogels in the collagenase-contained PBS at 37 °C **(E)** and equilibrium swelling properties of the hydrogels **(F)**.

When extruded through the printing nozzle, GelMA should have sufficient viscosity to create a hydrogel cylinder at the optimal printing temperature, but excessive viscosity would lead to severe shear stress. To find the ideal printing temperature, we examined the gelation temperature of each GelMA and GelMA/gelatin concentration (Fig. 3A,B). For bioprinting inks, the gelation temperature—the intersection of the G′ and G″ curves—is critical because it directly impacts scaffold quality. When the temperature is too high, GelMA liquefies, but when it’s too low, it overgels and becomes unprintable^26^. Pure GelMA at concentrations of 10%, 15%, and 20% had gelation temperatures of 26 °C, 29 °C, and 31 °C, respectively (Fig. 3A). Furthermore, with increasing concentrations of incorporated gelatin, the hybrid hydrogels’ storage modulus and loss modulus rose (Fig. 3B), indicating a correlation between higher moduli and concentration of incorporated gelatin.

The understanding of viscosity, shear thinning, and temperature interplay is fundamental for grasping the process of extruding hydrogels to fabricate intricate structures using 3D bioprinting. In Fig. 3C, the concentration of GelMA is observed to correlate with an increase in viscosity; conversely, higher temperatures lead to a reduction in viscosity across all samples. Specifically, at 15 °C, the 20% GelMA sample exhibited the highest viscosity, while the 10% GelMA group demonstrated the lowest viscosity at 25 °C. Additionally, the results illustrated a decrease in viscosity for each hydrogel as the shear rate increased (Fig. 3C,D). At a shear rate of 100 s^−1^, the viscosity of each group declined significantly, but it essentially returned to its original viscosity when the shear rate was reduced to 0.1 s^−1^. This suggests that every hydrogel group displays highly thixotropic behavior, a critical characteristic for extrusion-based 3D bioprinting.

The scaffold’s biodegradability is advantageous in tissue engineering as it eliminates the need for additional surgery to remove the implant. However, it is crucial that the rate at which the scaffold degrades coincides with the regeneration of new tissue. The scaffolds, comprising GelMA and GelMA/gelatin composites, were monitored for residual weights in PBS containing 1.25 U/mL type II collagenase for four weeks. Collagenase is a matrix metalloproteinase (MMP) enzyme that is capable of breaking down and rearranging the extracellular matrix to facilitate cell motility and spreading. Collagenase was employed in this study since it has been established that gelatin and GelMA include sequences that collagenases can identify^44^. The results indicate that the degradability rate reduces as the concentration of GelMA increases. GelMA at a concentration of 10% is completely destroyed after two weeks; however, for the concentration of 15% and the concentration of 20%, this degradation time spans three and four weeks, respectively (Fig. 3E). On the other side, adding gelatin to GelMA significantly reduces the breakdown rate, which is more noticeable at higher gelatin concentrations.

The swelling rate of the hydrogels is a critical feature because it directly influences the hydrogel’s ability to transport nutrients and metabolites, as well as its mechanical characteristic ^45^. Water permeability is also vital for cell adhesion and growth. According to Luangbudnark et al., a scaffold capable of absorbing fluid 80 times its original weight is suitable for skin tissue engineering^46^. This swelling can also be advantageous in the healing of wounds, as it creates a slight pressure on the edges of the wound^47^.

Figure 3F present the swelling ratio of hydrogels. The high levels of swelling (up to 8422.37%) demonstrates GelMA’s outstanding ability to absorb and retain water. According to this graph, increasing the GelMA concentration causes a decrease in swelling ratio, with GelMA 20% exhibiting the lowest ratio among the hydrogels. This decline in swelling ratio can be attributed to the reduction in pore diameter caused by the higher GelMA concentration, leading to decreased water uptake^42^. Moreover, the addition of gelatin to GelMA results in a significant reduction in the swelling ratio, with the extent of this reduction increasing with the gelatin content (Fig. 3F). The observed phenomenon can be explained by the fact that the inclusion of another biopolymer into GelMA fills up vacant spaces and restricts the transport of water within the GelMA matrix^48^. Our finding demonstrates that the extent of swelling in crosslinked GelMA gels could be regulated over a wide range by managing the GelMA concentration or the amount of gelatin that is added to GelMA. Further, comparing the weight loss and swelling ratio graphs leads to the conclusion that the more water absorbed by the hydrogel, the faster the scaffold degrades, and vice versa.

**Figure 4:**
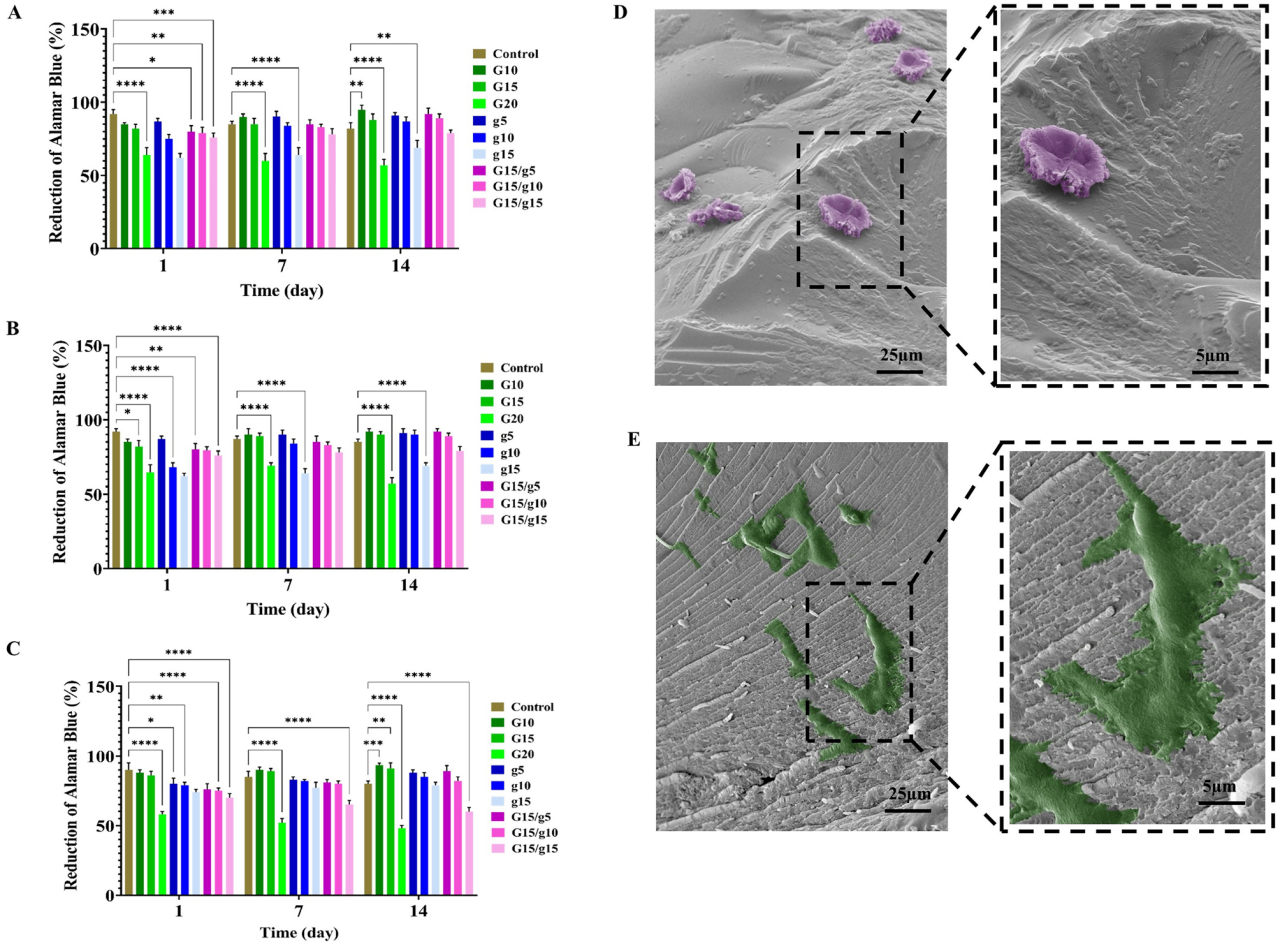
The Alamar blue test of the metabolic activity of keratinocytes **(A)**, fibroblasts **(B)**, and HUVECs **(C)** after 1, 7, and 14 days of culture in GelMA/gel hydrogels of varied formulations. SEM observation of the attachment and growth of HaCaT keratinocytes **(D)** and fibroblasts **(E)** on the GelMA/gelatin hydrogels. To improve visualization, cells in (**D**,**E**) are artificially colored.

According to the data from the Alamar blue assay, an increase in the concentration of gelatin at 15% and GelMA at 20% significantly reduces cell viability (Fig. 4A–C). It has been previously established that the pore size of gelatin scaffolds and cell proliferation are correlated^49^. Moreover, prior studies have indicated that higher GelMA concentrations lead to the formation of numerous covalent bonds, resulting in the scaffolds becoming more rigid and less porous, thereby diminishing the survival of HUVECs and HDFs within GelMA scaffolds^26,50^. These findings are consistent with those of our research, which showed a reduction in pore size from 100 µm in the G10 hydrogels, to 52 µm in the G20 hydrogels (Fig. 2C–F). The elastic modulus of the hydrogel also increased, from 14 kPa in the G10 and 32 kPa in the G15 to 78.4 kPa in the G20 (Fig. 2G). The results further suggest that the addition of gelatin to GelMA does not enhance cell growth, but it also does not reduce their viability (Fig. 4A–C). Considering that the incorporation of gelatin into GelMA has led to a decrease in pore size and an enhancement in mechanical strength, it can be inferred that gelatin has moderately improved the biocompatibility of GelMA.

SEM images show the adhesion and growth of keratinocytes (Fig. 4D) and fibroblasts (Fig. 4E) within the hydrogel. Keratinocytes exhibit a flat, nearly cubic morphology that closely resembles the cells found in the basal layer of the epidermis (Fig. 4D). Fibroblasts, on the other hand, have a morphology of stellate or spindle-shaped cells with long, slender processes that establish connections with the hydrogel, indicating that the GelMA/gelatin hydrogel provides favorable conditions for cell survival (Fig. 4E).

### Evaluation of cell behavior in GelMA/gelatin hydrogels containing AME

**Figure 5:**
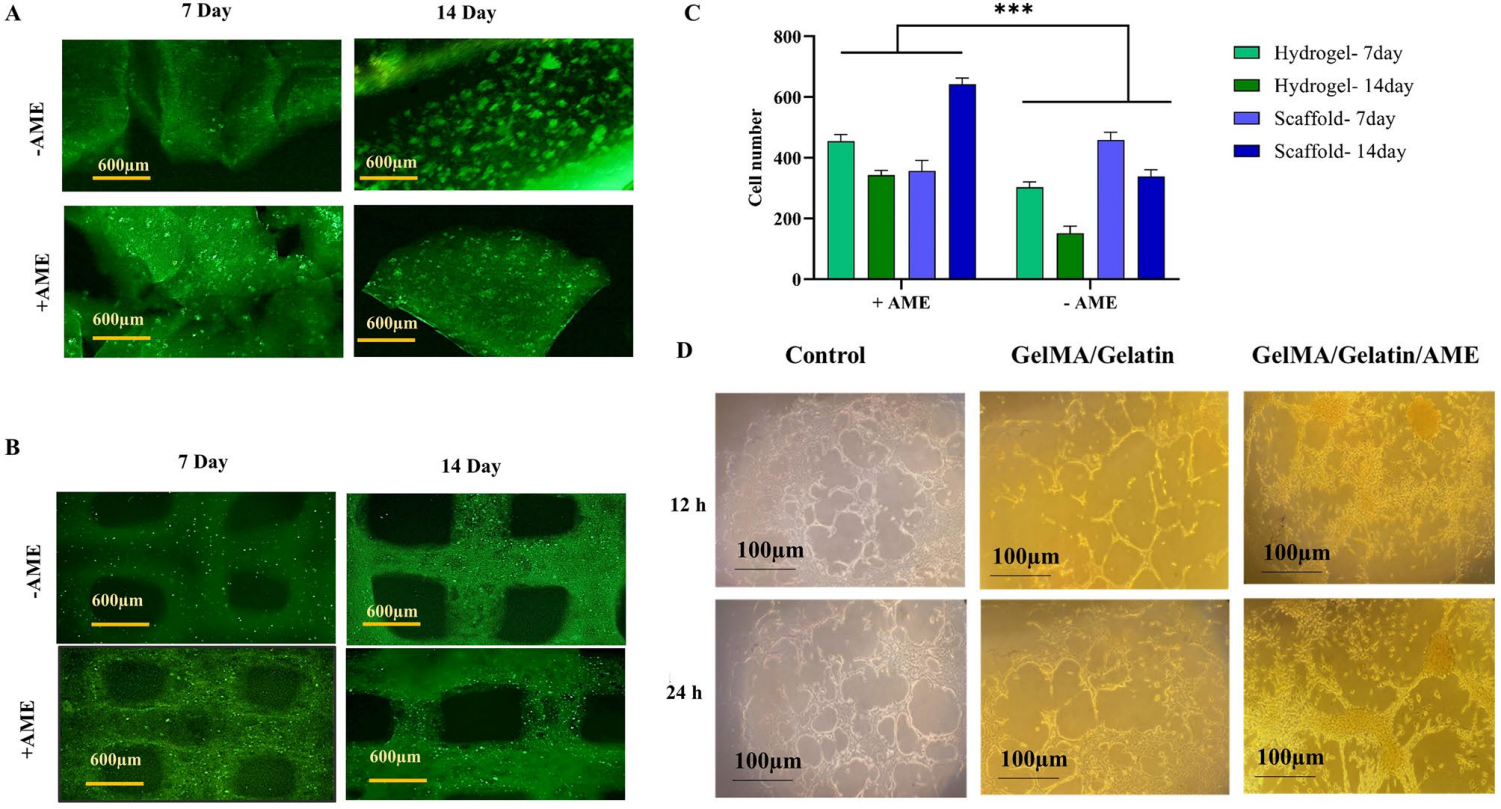
The viability assessment of HUVECs by live/dead staining, after culturing the cells in either the bulk hydrogel **(A)** or bio-printed scaffolds **(B)**. Quantification of the number of live cells using ImageJ software **(C)**. Tube formation assay for detecting the impact of GelMA/gelatin and AME on HUVECs’ rate of vascular network creation after 12 and 24 h of cell culture **(D)**.

To investigate the impact of AME on the growth of HUVECs, hydrogels and scaffolds containing HUVECs were cultured in the presence of AME. The viability of the cells was assessed after 7 and 14 days of cell culture using a staining kit (Fig. 5A,B). Quantification of viable cells with ImageJ software revealed that AME significantly enhanced cell growth in both hydrogels and scaffolds at the two time points (Fig. 5C).

The tube formation assay demonstrated that HUVECs formed tube-like structures on Matrigel (Fig. 5D). Even when HUVECs were cultured on GelMA/gelatin substrates, tube development was still observable. However, despite the increase in cell proliferation, the addition of AME to the GelMA/gelatin group seemed to interfere with tube formation (Supplementary Fig. 1).

**Figure 6:**
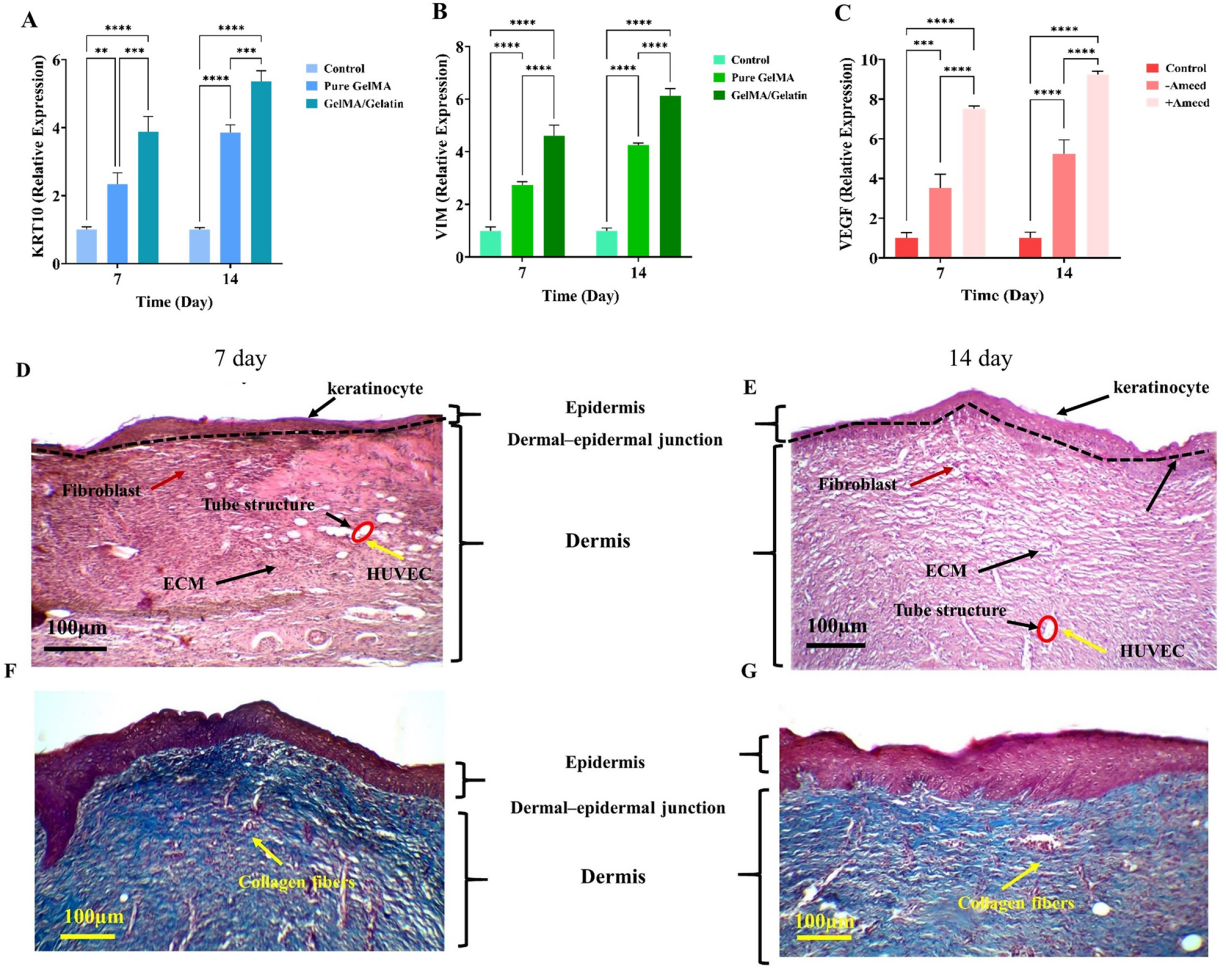
The gene expression of KRT10 in keratinocytes **(A)**, VIM in fibroblasts **(B)** and VEGF in HUVECs **(C)**. Vertical histology cross-section of the scaffolds stained with H&E at 7 day (D) and 14 day (E) as well as MT at 7 day **(F)** and 14 day **(G)**.

The impact of the amniotic membrane (AM) on angiogenesis remains a topic of debate, with conflicting reports in the literature. In a study by Bakhshandeh et al., it was found that exposure to human AME led to a decrease in the rate of HUVEC proliferation. Additionally, a reduction in the expression of von Willebrand factor and cluster of differentiation 31 was observed, suggesting inhibited vascularization. The authors attributed the results to the presence of anti-angiogenic factors within the amniotic membrane such as thrombospondin-1, endostatin, and heparin sulfate proteoglycan^51^. On the other hand, AME has been shown in several investigations to have a beneficial effect on angiogenesis. For example, Duan-Arnold et al. discovered that AME promotes angiogenesis in chronic lesions. The authors proposed that the enhanced angiogenesis activity could be attributed to elevated levels of VEGF observed in cryopreserved AM^52^. In another study, by evaluating angiogenic potential of AM using the rat aortic ring assay and microvessel imaging, Niknejad et al. found that AM devoid of amniotic epithelial cells can stimulate angiogenesi ^53^. Niknejad and Yazdanpanah explained the reported discrepancies by arguing that the epithelial side of the AM restricts vessel sprouting and decreases capillary numbers, but the mesenchymal side of the AM promotes angiogenesis^54^. The previous study^34^, from which we derived the AME extraction process, found that HAMs contain significant levels of epidermal growth factor (EGF) and keratinocyte growth factor (KGF), which are most likely secreted by epithelial cells. As a result, it may be inferred that the presence of these growth factors has increased the quantity of HUVECs while perhaps reducing their ability to form tubes. On the other hand, the middle layer of the amniotic membrane contains pro-angiogenic growth factors such as VEGF^55^. Our research has also demonstrated that supplementing GelMA/gelatin scaffold with AME enhances the expression of the VEGF gene in HUVECs (Fig. 6C). The variety and quantity of biological components in the amniotic membrane seem to be linked to different angiogenesis outcomes. Future studies could investigate the specific effects of each component of the amniotic membrane on angiogenesis, including extracts from de-epithelialized amniotic membrane.

### Gene expression analysis

The qRT-PCR analysis indicates a statistically significant upregulation in the expression of the KRT gene in keratinocytes (Fig. 6A) and VIM in fibroblasts (Fig. 6B) when gelatin was added to the GelMA scaffolds, and VEGF in HUVECs when cultured in scaffolds containing AME (Fig. 6C), as compared to the control group. Previous studies have indicated that both gelatin and GelMA can promote the proliferation and differentiation of dermal cells by establishing a moisture-rich setting and providing structural resemblances to the ECM^43^. By immobilizing gelatin on the surfaces of PCL nanofibers, Pham-Nguyen et al. showed increased expression of keratinocyte-specific genes during dorsal wound healing in mice^56^. It is worth mentioning that although gelatin and GelMA share a substantial chemical similarity, even minute variations in the type, amount, and positioning of their functional groups might affect which genes are expressed and to what degree in the encapsulated cells. Notably, NMR test results indicated that gelatin possesses more amino and hydroxyl functional groups than GelMA (Fig. 1A). These functional groups can substantially influence the osteogenic or chondrogenic differentiation of stem cells^57^. Additionally, through the modulation of integrin binding, they have the ability to control focal adhesion composition, assembly, and signaling^58^. Additional research is needed to ascertain the exact function of each functional group in the gene expression of fibroblasts and keratinocytes.

### Histological analysis of bioprinted GelMA/gelatin/AME scaffolds cultured with keratinocytes, fibroblasts, and HUVECs

Cell morphology and ECM production of keratinocytes, fibroblasts, and HUVECs were examined after 7 and 14 days of culture in GelMA/gelatin scaffolds with or without AME. Histological staining was performed to analyze the results. H&E staining illustrates the development of distinct skin layers in GelMA\gelatin hydrogels after 14 days of cells culture compared to 7 days (Fig. 6D,E). The differentiation line between the two layers is fully formed, with keratinocyte cells in the upper layer and fibroblast cells in the lower layer. H&E staining also demonstrates a uniform distribution of cells in all GelMA/gelatin hydrogels. Fibroblasts were identified as elongated cells with cytoplasmic extensions (marked by red arrows), while HUVECs appeared as round or cobblestone-shaped cells (indicated by yellow arrows) in the images. In addition, HUVEs have developed tube-like structures, which are indicated by a red circle in the Fig. 6D,E. Both fibroblasts and HUVECs maintained their morphology, indicating their viability and successful three-dimensional attachment to the substrate. MT staining was utilized to emphasize the development of collagen fibers of connective tissue in the scaffolds (Fig. 6F,G). In this staining, collagen fibers appeared blue-green, with the intensity of this color correlating to the amount of deposited collagen. This intensity notably increased after 14 days of cells culture in the lower section of the scaffold, indicating the formation of connective tissue fibers by fibroblast cells. Overall, histological staining suggests that the scaffolds create a suitable environment for skin cells activity and ECM production, particularly in the presence of AME.

## Conclusion

The study aimed to develop a scaffold for skin tissue engineering using a 3D-printed GelMA/gelatin composite material. In this work, the upper stratum of the scaffold was designated for cultivating keratinocytes, while the lower part was used to cultivate fibroblasts and HUVECs, aiming to mimic the structure of natural skin. Mechanical and degradability studies indicated that adding gelatin to GelMA improves its physical qualities. Survival and cell proliferation tests affirmed the scaffold’s biocompatibility. In addition, real-time PCR analysis demonstrated that adding gelatin to GelMA increases the expression of VIM in fibroblast and KRT10 in keratinocytes, and also the presence of AME enhances the expression of VEGF in HUVECs. Further, the histological staining of the scaffolds revealed well-defined strata, within which cells retained their shape, and the extracellular matrix had successfully formed. Based on these results, it appears that the developed scaffold holds potential for use in skin tissue engineering applications. One drawback of our work was the absence of supplementary growth factors to enhance keratinocyte differentiation. A more realistic replication of the skin’s natural structure may be achievable through the careful incorporation of specific growth factors at appropriate locations and concentrations.

### Supplementary Information

Supplementary Figure 1.

## Data Availability

The datasets used in the present study are available from the corresponding author on reasonable request.
