# Supplementary material for: 3D-bioprinted GelMA/gelatin/amniotic membrane extract (AME) scaffold loaded with keratinocytes, fibroblasts, and endothelial cells for skin tissue engineering
Source: Sci Rep. 2024 Jun 3;14:12670. doi: 10.1038/s41598-024-62926-y (PMC11148016; doi:10.1038/s41598-024-62926-y)
Supplement: Supplementary file 1 — Supplementary Figure 1. [file 41598_2024_62926_MOESM1_ESM.docx]

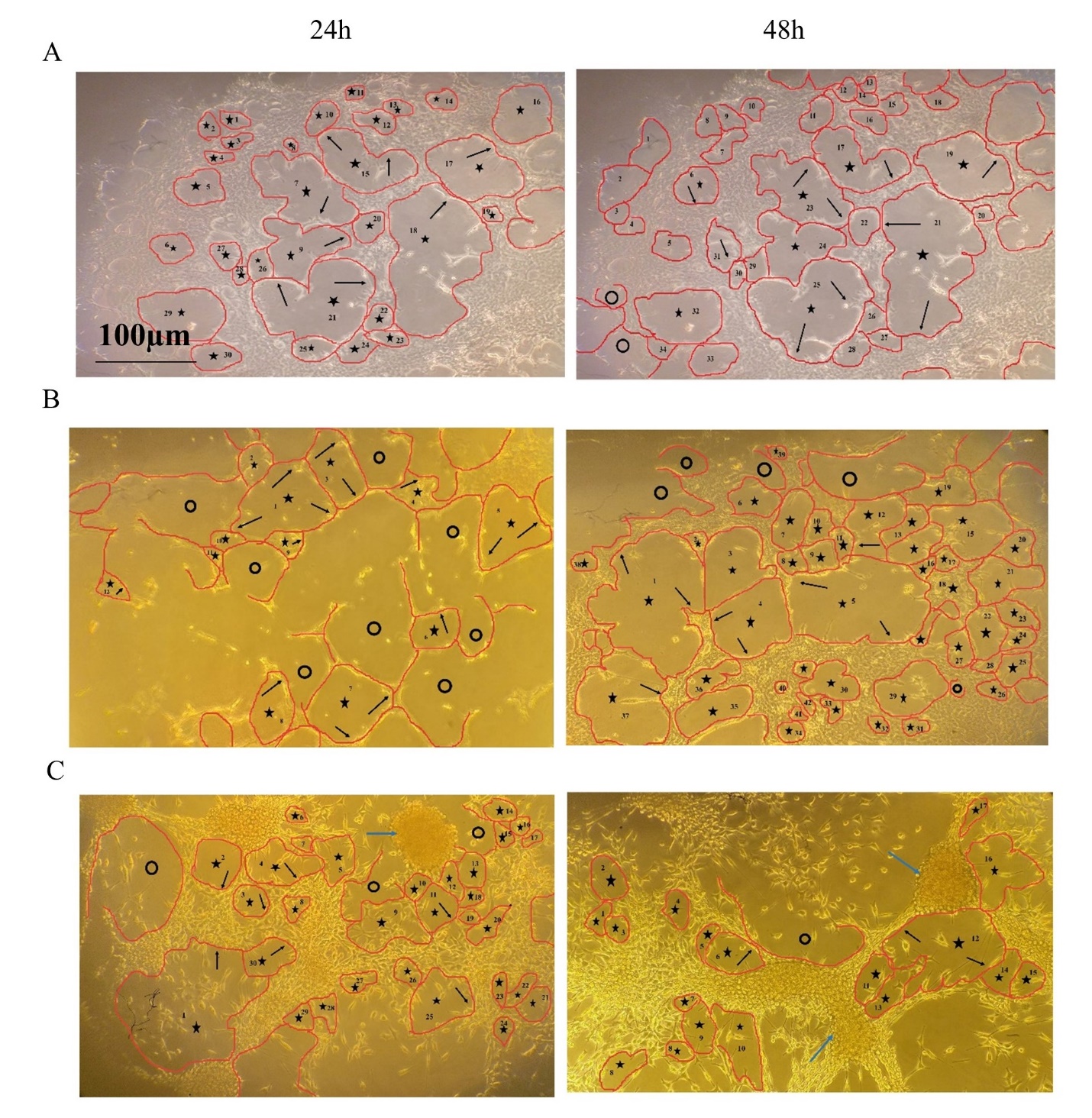


Figure 1.S Tube formation assay for detecting the impact of Matrigel **(A)**, GelMA/gelatin **(B)**, and GelMA/gelatin/AME **(C)** on HUVECs' rate of vascular network creation after 12 and 24 hours of cell culture.

In this figure, the endothelium-derived tubes are numbered within the tubes. Semi-closed circles are indicated with O, and whole circles with *. In addition, black arrows highlight junctions and branches, while blue arrows show areas of significant cell proliferation.
